# Can AI Predict the Magnitude and Direction of Ortho-K Contact Lens Decentration to Limit Induced HOAs and Astigmatism?

**DOI:** 10.3390/jcm13185420

**Published:** 2024-09-12

**Authors:** Wen-Pin Lin, Lo-Yu Wu, Wen-Kai Li, Wei-Ren Lin, Richard Wu, Lynn White, Rowan Abass, Rami Alanazi, Joseph Towler, Jay Davies, Ahmed Abass

**Affiliations:** 1 Department of Optometry, University of Kang Ning, Taipei 11485, Taiwan; 2 Research and Development Centre, Brighten Optix Corporation, Taipei 111, Taiwan; 3 Department of Optometry, Mackay Medical College, New Taipei 252, Taiwan; 4 Department of Power Mechanical Engineering, Nation Tsing Hua University, Hsinchu 300, Taiwan; 5 College of Optometry, Pacific University, Forest Grove, OR 97116, USA; 6 Research and Development Department, LWVision, Leicester LE18 1DF, UK; 7 Wirral Grammar School for Girls, Bebington CH63 3AF, UK; 8 Department of Materials, Design and Manufacturing Engineering, School of Engineering, University of Liverpool, Liverpool L69 3GH, UK; 9 Department of Eye and Vision, Institute of Life Course and Medical Sciences, University of Liverpool, Liverpool L7 8TX, UK

**Keywords:** ortho-k, contact lenses, decentration, HOAs, astigmatism, AI

## Abstract

**Background**: The aim is to investigate induced higher-order aberrations (HOA)s and astigmatism as a result of non-toric ortho-k lens decentration and utilise artificial intelligence (AI) to predict its magnitude and direction. **Methods:** Medmont E300 Video topographer was used to scan 249 corneas before and after ortho-k wear. Custom-built MATLAB codes extracted topography data and determined lens decentration from the boundary and midpoint of the central flattened treatment zone (TZ). An evaluation was carried out by conducting Zernike polynomial fittings via a computer-coded digital signal processing procedure. Finally, an AI-based machine learning neural network algorithm was developed to predict the direction and magnitude of TZ decentration. **Results:** Analysis of the first 21 Zernike polynomial coefficients indicate that the four low-order and four higher-order aberration terms were changed significantly by ortho-k wear. While baseline astigmatism was not correlated with lens decentration (R = 0.09), post-ortho-k astigmatism was moderately correlated with decentration (R = 0.38) and the difference in astigmatism (R = 0.3). Decentration was classified into three groups: ≤0.50 mm, reduced astigmatism by −0.9 ± 1 D; 0.5~1 mm, increased astigmatism by 0.8 ± 0.1 D; >1 mm, increased astigmatism by 2.7 ± 1.6 D and over 50% of lenses were decentred >0.5 mm. For lenses decentred >1 mm, 29.8% of right and 42.7% of left lenses decentred temporal-inferiorly and 13.7% of right and 9.4% of left lenses decentred temporal-superiorly. AI-based prediction successfully identified the decentration direction with accuracies of 70.2% for right and 71.8% for left lenses and predicted the magnitude of decentration with root-mean-square (RMS) of 0.31 mm and 0.25 mm for right and left eyes, respectively. **Conclusions:** Ortho-k lens decentration is common when fitting non-toric ortho-k lenses, resulting in induced HOAs and astigmatism, with the magnitude being related to the amount of decentration. AI-based algorithms can effectively predict decentration, potentially allowing for better control over ortho-k fitting and, thus, preferred clinical outcomes.

## 1. Introduction

**Figure 1:**
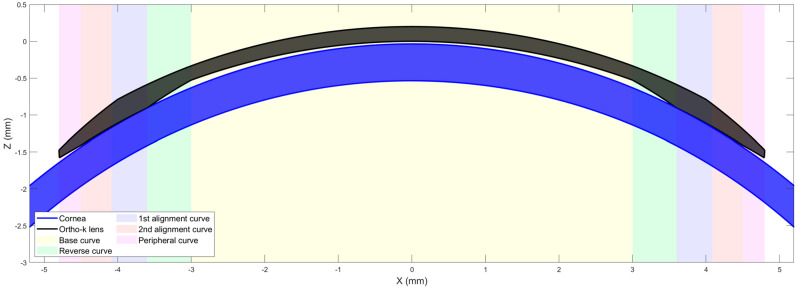
Centred ortho-k lens on a cornea.

**Figure 2:**
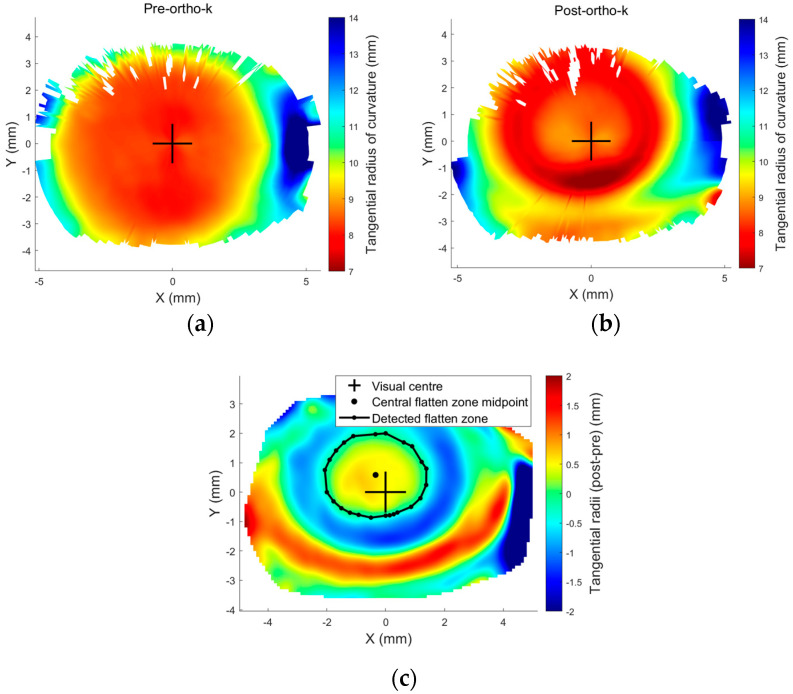
Tangential radii of curvature (**a**) pre-ortho-k wear, (**b**) post ortho-k wear, and (**c**) the smoothed difference maps showing a decentred treatment zone produced by a decentred ortho-k lens. Row height data were exported from Medmont E300 topographer for a 19-year-old male subject and then processed via a custom-built MATLAB code.

Modern orthokeratology (ortho-k) designs [1,2], intended for use for either the temporary reduction of myopia or for myopia control, differ from conventional rigid gas-permeable (RGP) contact lenses in that they are a “reverse geometry” design having a central base curve that is flatter than the natural curvature of the cornea and a peripheral design that aligns with the peripheral cornea [3]. The intervening zone is termed a “reverse curve” and has a curvature steeper than the cornea, Figure 1. A well-centred ortho-k contact lens has the effect of flattening the central cornea, creating a treatment zone (TZ) over the pupil while creating a raised annulus around the TZ, resulting in the typical bullseye pattern as seen on topography difference maps, Figure 2, where the baseline map is compared to the post-treatment map. Ortho-k lenses are generally prescribed for overnight wear so that the patient can see normally, lens-free, during the day. For myopia management, the intention is to temporarily minimise the patient’s myopic refractive error as far as possible during daytime. For myopia control, there is an additional intention to slow the progress of myopia by stabilising axial length growth.

The mechanical effect of ortho-k on the cornea is to thin the central cornea within the TZ and cause the surrounding tissue to be thickened or raised [4]. These changes in shape and thickness lead to induced HOAs [5] and changes in peripheral refraction, which have been linked to the slowing of myopia progression [6,7,8,9,10], although some studies show less or no correlation between induced HOAs and the slowing of axial length growth [11]. The variability in study results may be linked to a lack of control over the centration of ortho-k lenses; a decentred lens will affect the corneal shape and thickness profile, which in turn will affect induced HOAs and peripheral refraction [12] as well as contrast sensitivity [13]. Ortho-k contact lens centration, therefore, is essential regarding its effect on HOAs, which in turn has an impact on myopia control [14,15,16,17], and it can be assessed by examining the TZ decentration (TZD) in pre- to post-ortho-k tangential difference maps, Figure 2. Additionally, it is crucial to understand the link between the amount of decentration and induced HOAs. This has valued clinical usage in attempting to balance the potential benefits of induced HOAs in terms of the effects on myopia progression versus the downside of visual symptoms of ghosting and haloes and the reduction in contrast sensitivity.

Chen et al. [18] attempted to predict ortho-k lens decentration with corneal elevation in 36 eyes via an oversimplified method using corneal elevation asymmetry that statistically and strongly correlated decentration direction to corneal asymmetry. Considering the relatively small sample size and shortening all corneal elevation meridians to a single asymmetry vector, the limited reports in the literature agreed on this simple assessment. While investigating the influence of overnight orthokeratology lens fitting decentration on corneal topography reshaping on 106 eyes, Chen et al. [19] reported temporal-inferior decentration among 49.1% of fitted ortho-k lenses. Gu et al. [20] considered the influence of corneal topographic parameters in the decentration of ortho-k in 50 eyes and reported temporal-inferior decentration in 72% of them. The common feature of these studies was that they simplified the corneal surface and considered asymmetries in different ways. This study, in contrast, considers the whole anterior corneal surface in decentration prediction analysis.

This study investigates the effect and magnitude of decentration on the resultant HOAs of the cornea and specifically on irregular astigmatism. AI is utilised to determine if it is possible to predict TZD, and thus ortho-k contact lens decentration, from the lens parameters and the corneal shape.

## 2. Methods

### 2.1. Topography Data Collection and Processing

The study utilises fully anonymised records from 249 eyes, 132 right eyes and 117 left eyes, selected for solely secondary analysis processing, Table 1. The clinical data collection was approved by the ethical committee of the institutional review board of Taipei Medical University in Taiwan (N201810065/23-11-2018) and conducted following the standards set in the Declaration of Helsinki. Patients were instructed to wear their lenses overnight for at least a week and revisit the clinic for a first follow-up, with a second follow-up a month later and then at consistent intervals. The current study only considered patients who wore lenses for between 10 and 100 days. Intraocular pressure (IOP) and central corneal thickness (CCT) were recorded at the initial visit using Non-Contact Tonometer/Pachymeter TONOPACHY™ NT-530P (Nidek Co., Ltd., Gamagori, Japan).

Medmont E300 height files “*.hgt” and distance files “*.dst” were extracted and automatically scanned with a custom-built MATLAB code where a grid of 300 angular positions and 333 radial positions were used to construct the corneal anterior surface using the corneal height.

### 2.2. Ortho-K Contact Lens Decentration Analyses

Ortho-k contact lens decentration was determined by investigating the TZD (resultant central flattened area) following the method described in [21], using the tangential power difference map between pre- and post-ortho-k lens wear. Then, to allow a common coordinate among pre- and post-maps, 3D triangulation-based cubic interpolation [22] was used to reconstruct the post-ortho-k corneal surface to ensure it was mesh-aligned with the pre-ortho-k wear corneal surface. To avoid any potential influence caused by a built-in signal processing element within the topographer software, the analysis in this study did not rely on the map output of the Medmont E300 software package (7.2.9). Instead, a custom-built MATLAB function was used to determine the tangential power from the raw data. With a Cartesian coordinates grid covering the range −6 to 6 mm in both the X direction (nasal–temporal) and the Y direction (superior–inferior), the Z axis represented the anterior eye surface height. Radial direction distance was defined by $\rho_{g}$, Equation (1), where

(1) $$\rho_{g}=\sqrt{{X_{g}}^{2}+{Y_{g}}^{2}},$$

where $X_{g}$ and $Y_{g}$ characterise the coordinates of each of the grid points. The tangential curvature $K_{t}$ and its radius $R_{t}$ can be expressed by the first and second derivatives of the height *Z* relative to the radial distance through the differential equation, Equation (2).

(2) $$K_{t}=\frac{1}{R_{t}}=\frac{d^{2}Z_{g}/d{\rho_{g}}^{2}}{{\left(1+{\left(dZ_{g}/d\rho_{g}\right)}^{2}\right)}^{\frac{3}{2}}}$$

The corneal net refractive power $P_{net}$ was typically determined using the Gaussian optics formula [23,24], Equation (3), as

(3) $$P_{net}=\frac{n_{cornea}-n_{air}}{R_{anterior}}+\frac{n_{aqueous}-n_{cornea}}{R_{posterior}}-\frac{CCT}{n_{cornea}}\times \frac{n_{cornea}-n_{air}}{R_{anterior}}\times \frac{n_{aqueous\;}-n_{cornea}}{R_{posterior}}$$

where the refractive indices of air, $n_{air}$, cornea, $n_{cornea}$, and aqueous, $n_{aqueous}$, are set to 1.0, 1.376 and 1.336, respectively, following Gullstrand’s relaxed eye model [25,26]. As Medmont E300, like other Placido topographers, does not measure the corneal posterior surface or the central corneal thickness CCT, only the anterior radii of curvature $R_{anterior}$ can be used to determine corneal net refractive power approximately. In this case, $R_{anterior}$ was set to $R_{t}$ and Equation (3) was reduced to Equation (4), where $n_{eq}$ was set to a hypothetical value of $n_{h}=1.3375$, as it worked as a correction element compensating for the absence of the posterior corneal refractive component. Hence, the tangential refractive power $P_{t}$ could be expressed as in Equation (4).

(4) $$P_{t}=\frac{n_{h}-n_{air}}{R_{t}}$$

At this stage, the difference between the corneal post-ortho-k wear tangential refractive power and pre-ortho-k wear tangential refractive power, $∆P_{t}$, could then be established as a map according to Equation (5).

(5) $$∆P_{t}={P_{t_{post}}-P}_{t_{pre}}\;\mathrm{where}\;P_{t_{pre}}=\frac{n_{h}-n_{air}}{R_{t_{pre}}}\;\&\;P_{t_{post}}=\frac{n_{h}-n_{air}}{R_{t_{post}}}$$

Once the power difference map of $∆P_{t}$ was obtained, the central flattened zone was automatically detected by locating the intersection between the pre- and post-ortho-k tangential refractive power at each meridian, Figure 2c. Then, the resulting scattered points were fitted to a circle using the least root mean square error method. The TZ decentration was measured by locating the geometrical centre of the circular fitted boundary of the central flattened zone relative to the corneal apex, represented by the radii measured from the cornea centre and angles measured from the nasal side axis. The Cartesian coordinate system could be broken into four cardinal quadrants by the X-axis (nasal–temporal) and the Y-axis (superior–inferior). For right eyes, quarter 1 is the nasal-superior side, quarter 2 is the temporal-superior side, quarter 3 is the temporal-inferior side, and quarter 4 is the nasal inferior side. For left eyes, quarter 1 is the temporal-superior side, quarter 2 is the nasal-superior side, quarter 3 is the nasal-inferior side, and quarter 4 is the temporal-inferior side. These four quarters were later used for classification when AI algorithms were applied and called classes 1 to 4. Right and left eyes were treated independently at this stage to prevent possible bias in the findings [27,28].

### 2.3. Zernike Polynomial Analyses

Zernike established a sequence of orthogonal polynomial functions of two variables, unit circle limited radius (ρ) and angular position (φ) [29]. Using Zernike polynomials, the corneal surface can be constructed from a combination of terms with a physical meaning directly linked to the ocular surface characteristics when fitted to corneas [30]. A normalised form ρ of the radius $\rho_{g}$ is required for a Zernike polynomial fit [31], and can be calculated as

(6) $$\rho =\frac{\rho_{g}}{\rho_{max}},$$

where $\rho_{max}$ is the maximum radius noted in the topography data, which in this case was set to 5 mm to ensure that the data were in Medmont’s most reliable measurement area, as peripheral measurements are less reliable because of the edge effect. Any surface data beyond this maximum radius were disregarded in these analyses. The Zernike raw elevation $Z_{n}^{m}(\rho ,\varphi )$ is given by [29]

(7) $$Z_{n}^{m}\left(\rho ,\varphi \right)=\left\{\begin{array}{cc} R_{n}^{m}\cos\left(m\varphi \right) & m>0\\ R_{n}^{m}\sin\left(m\varphi \right) & m<0 \end{array}\right.,$$

where φ is the azimuthal angle corresponding to the coordinates $X_{g}$ and $Y_{g}$, n is the radial order of the polynomial, m an azimuthal integer index that changes from −n to n for even (m − n) and equals 0 for odd (n − m), and $R_{n}^{m}$ is a radial polynomial defined as

(8) $$R_{n}^{m}\left(\rho \right)=\sum_{k=0}^{\frac{n-m}{2}}\frac{{\left(-1\right)}^{k}(n-i)!\rho^{n-2k}}{k!((n+m)/2-k)!((n-m)/2)!}\;\;\left(0\leq \rho \leq 1\right).$$

The Zernike raw elevation (height) term $Z_{n}^{m}(\rho ,\varphi )$ was fitted to the anterior corneal surfaces exported by the MATLAB(R2024a)-based custom-built software. The root-mean-square (RMS) error was calculated for every fit as

(9) $$RMS=\sqrt{\frac{\sum_{i=1}^{q}{\left(Z_{i\;fit}-Z_{i\;surf}\right)}^{2}}{N}},$$

where $Z_{fit}$ is the Zernike fitted surface height, $Z_{surf}$ is the measured surface height, and N is the total number of data points used for the RMS calculation. During the fitting process, 80% of the data points were randomly picked for polynomial fitting, and the remaining 20% were used for the RMS error estimate, following the Pareto principle [32]. 

### 2.4. Astigmatism Quantification Analyses

The current study focuses on corneal astigmatism, assuming that any crystalline lens astigmatism (lenticular astigmatism) was constant for the same subject between the pre- and post-ortho-k topography measurements. Taking into account the variable sensitivity of different methods that map corneal refractive power [33,34], a tangential refractive power map was established for each eye using a custom-built MATLAB code; hence, the central 3 mm diameter was considered, and then flat and steep power meridians were automatically identified with a 1° angular step, covering the central area of the cornea. Centres of power maps were assumed to lie on the corneal visual axis [35]; therefore, the difference between these powers was taken as a measure of corneal astigmatism.

### 2.5. Violin Plot Representation of Induced Astigmatism

Violin plot representation was used in the current study as, unlike a traditional box plot, it provides a comprehensive view of the data by combining a box plot’s summary statistics with a kernel density plot’s density information [36]. In the current study, the violin plot shows the distribution of the decentration data across three different categories (less or equal to 0.5 mm, between 0.5 to 1.0 mm and greater than 1.0 mm), with the density of the data characterised by the width of the violin form. The central horizontal line displays the mean and median summary statistics, while the density plot flanks the category vertical line on either side, showing the data distribution. The width of the violin at any given vertical axis value corresponds to the normalised data density at that value. Finally, the horizontal axis represents the three categories being compared.

### 2.6. Use of AI to Predict TZD and Ortho-K Lens Decentration

The current study created a 10-hidden-layer feedforward artificial intelligence (AI) neural network structure with training and validation ratios of 70% and 30%, respectively. The input layer receives the feature variables that describe the data, and subsequently, hidden layers perform computations on the input layer’s data through trained biased weighted connections between calculation stations, called nodes. Each node smears an activation function to the weighted sum of its inputs, introducing nonlinearity and enabling the network to develop complex combinations. Finally, the output layer generates the predicted values based on the computations achieved in the hidden layers. After training, the neural network predicts unseen test data by feeding the input features through the trained network, and the output layer provides the predicted continuous values [37,38,39,40].

Activation transfer functions were set to Tan-Sigmoid transform for hidden layers and linear transform for the output layer while setting the learning rate to 0.01 and training epochs to 100 using the “fitnet” and “train” MATLAB’s Neural Network Toolbox functions. In Medmont E300, corneal height data were re-meshed with radii ranging from 0 to 5 mm in 10° steps into polar coordinates. In this context, polar coordinates are a two-dimensional coordinate system in which each point is identified by a distance from the origin and an angle from a datum direction. The reference point is called the pole, analogous to the corneal centre, and the reference direction is typically the nasal side axis. Then, polar corneal height data formed the input, while decentration Cartesian quarter (1 to 4) and decentration radii were the AI-model outputs. Finally, the established AI machine learning neural network algorithms predicted decentration direction and radii.

### 2.7. Statistical Analysis

The statistical analyses conducted on the outcomes of this study were carried out utilising MATLAB software’s Statistics and Machine Learning Toolbox. A null hypothesis, set to 95.0% confidence level, was used to examine the inferences of the findings based on statistical indication. The normality of the samples’ distribution was confirmed using the Kolmogorov–Smirnov test via MATLAB [41]. In order to compare two sets of results, the two-sample *t*-test was employed to examine whether there was statistical significance between data sets and whether the assessed findings showed independent behaviour. The probability rate (p-value) ranged from 0.0 to 1.0, where *p* < 0.05 implies the invalidity of the null hypothesis, hence significance [42]. The MATLAB function ‘ttest2’ and the returned *p*-value and binary test decision for the null hypothesis were used. The correlation coefficient used in this study (R) measures the linear dependence of two variables [43]. R values below 0.3 indicate weak correlations; R values in the range of 0.3 to 0.7 indicate moderate correlations; and finally, R values above 0.7 indicate strong correlations [44].

## 3. Results

### 3.1. Zernike Polynomial Fitting

**Figure 3:**
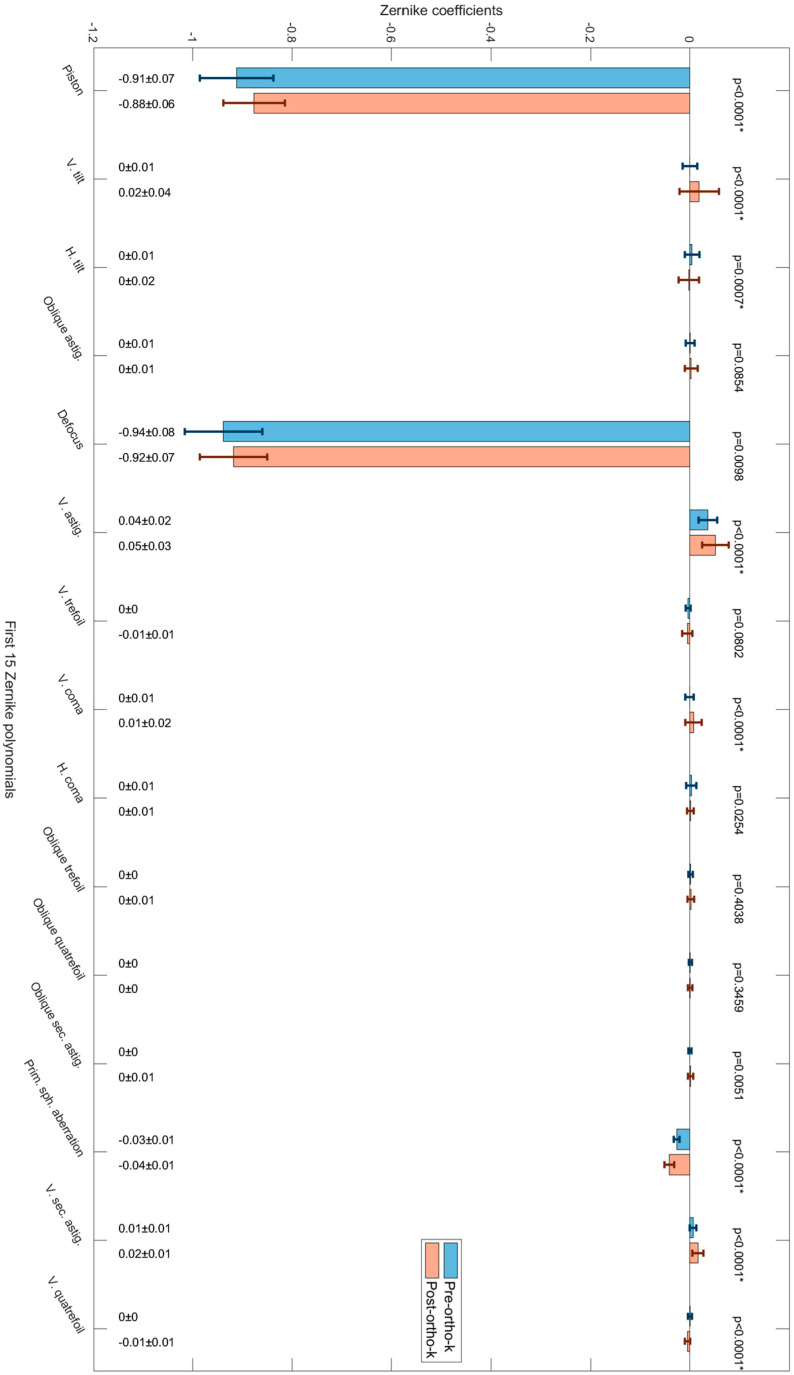
The first 15 Zernike polynomials and their pre- and post-ortho-k wear coefficients with standard deviation represented by error bars and *p*-values of less than 0.05 indicate statistically significant differences.

When Zernike polynomials were unlisted for investigating the pre- and post-ortho-k wear (Figure 3), terms such as “piston” showed significant reductions in their magnitude (*p* < 0.0001), as expected from such treatment; however, terms such as “vertical tilt”, “horizontal tilt”, “vertical astigmatism”, “vertical comma”, “primary spherical aberration” and “vertical secondary astigmatism” also showed significant changes in their magnitude (*p* < 0.0001), Table 2.

**Figure 4:**
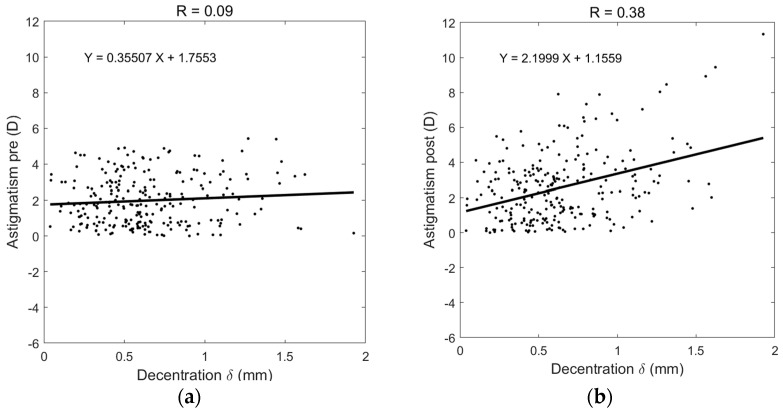
Decentration correlation with astigmatism in (**a**) pre-ortho-k wear, (**b**) post-ortho-k wear.

**Figure 5:**
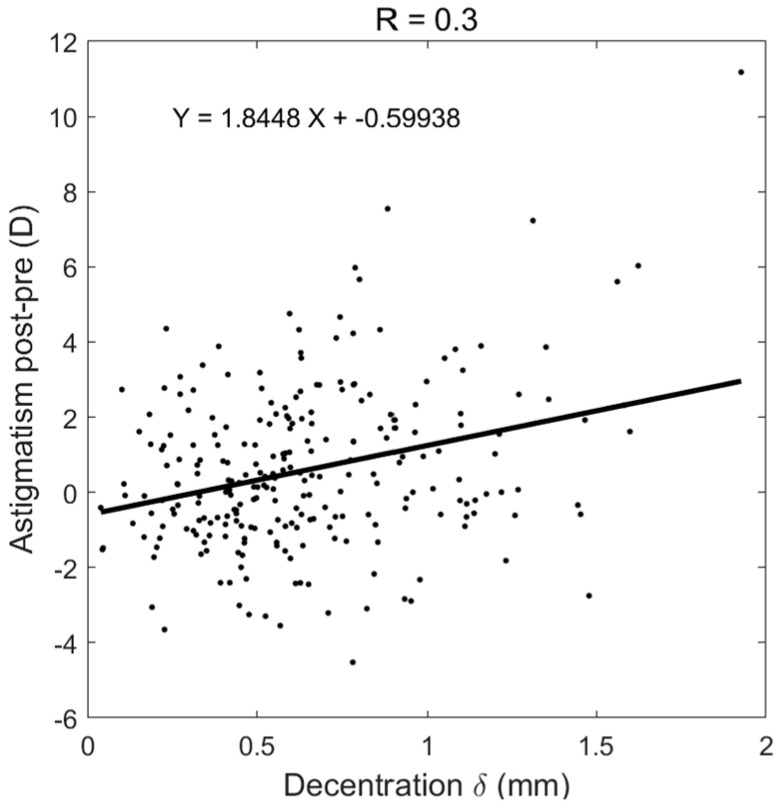
Increased astigmatism after ortho-k wear correlated with post-ortho-k recorded TZD.

In order to evaluate induced astigmatism caused by decentred ortho-k lenses, the amount of post-ortho-k TZD was plotted against the pre-ortho-K astigmatism, Figure 4a, and post-ortho-k astigmatism, Figure 4b. While nearly no correlation was recorded between decentration and pre-ortho-K astigmatism (R = 0.09), a moderate correlation (R = 0.38) was observed when the post-ortho-K wear TZD was compared against post-ortho-K astigmatism. To evaluate induced astigmatism due to ortho-k lens wear, the change in astigmatism power post-ortho-k minus pre-ortho-k was calculated and showed a moderate correlation (R = 0.3) with decentration, Figure 5.

### 3.2. Decentration Assessment

**Figure 6:**
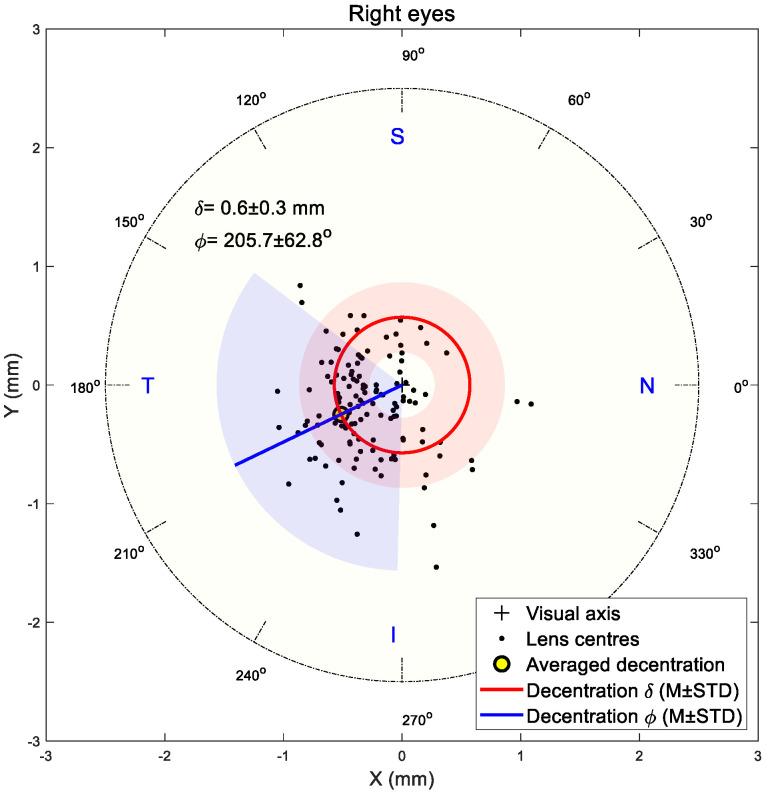
Flattened central zone position on right eyes. Light red and blue areas represent standard deviations of radii and angles, respectively.

**Figure 7:**
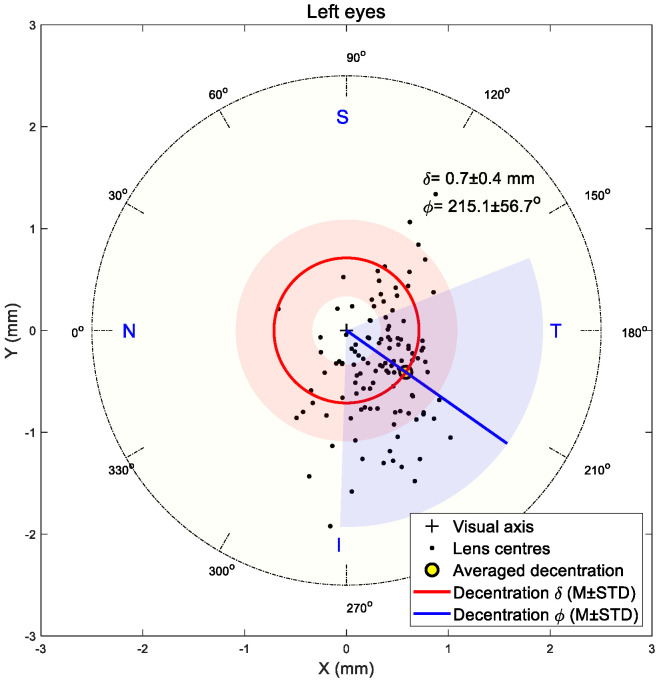
Flattened central zone position on left eyes. Light red and blue areas represent standard deviations of radii and angles, respectively.

The shift in the flattened central zone midpoint was utilised to indicate the TZD in the current study following the research team’s previous work [3,45]. Results showed that decentration in both right and left eyes was towards the inferior nasal area in most cases. The right eyes’ central flattened zone recorded 0.6 ± 0.3 mm average decentration with a 205.7 ± 62.8° counterclockwise angle from the nasal side, Figure 6, while the left eyes recorded 0.7 ± 0.4 mm average decentration with a 215.1 ± 56.7° clockwise angle from the nasal side, Figure 7.

**Figure 8:**
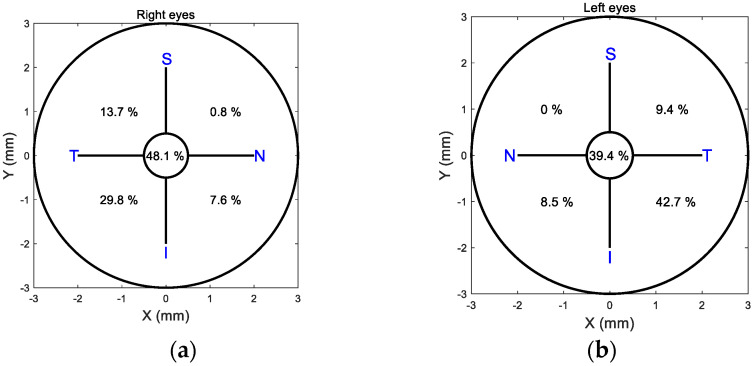
Percentages of Ortho-k lens centration in each quadrant show that more than 50% of lenses were decentred more than 0.5 mm. Right eyes are represented in (**a**), and left eyes are represented in (**b**).

When a 0.5 mm decentration limit was taken as a threshold for centration, and the cornea was divided into four quarters, 48.1% of right eye lenses were centred, Figure 8a, while 39.4% of left eye lenses were centred, Figure 8b. The temporal inferior side attracted 29.8% of right lens decentrations and 42.7% of left lens decentrations. The second highest decentration quarter was the temporal superior side, with 13.7% right lenses and 9.4% left lenses decentred.

**Figure 9:**
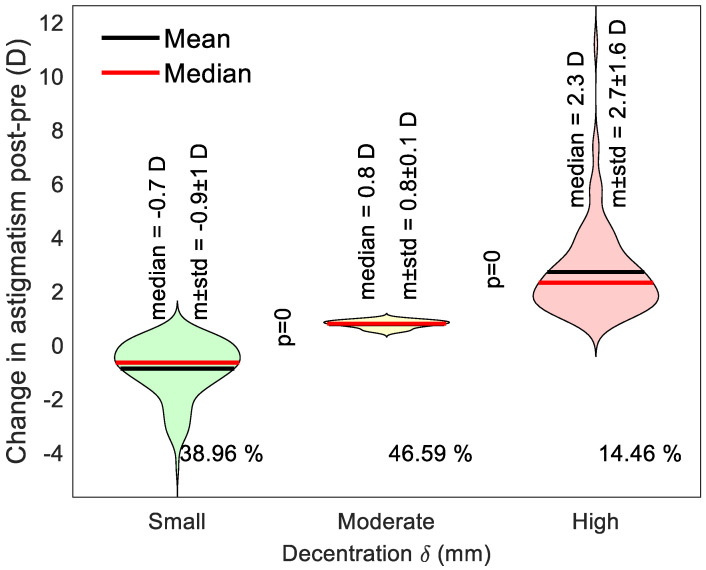
A violin plot showing the distribution of the change in astigmatism among three categories of decentration: small decentration (up to 0.5 mm), moderate decentration (over 0.5 to 1.0 mm) and high decentration (over 1.0 mm).

An overall investigation of decentration in both right and left eyes explored the link between ortho-k lens decentration and the change in three categories of decentration: small decentration (up to 0.5 mm), moderate decentration (over 0.5 to 1.0 mm) and large decentration (over 1.0 mm). Results showed that around 39% of eyes had a small decentration and recorded an average reduction in astigmatism of −0.9 ± 1 D, 46.59% of eyes had a moderate decentration and recorded an average increase in astigmatism of 0.8 ± 1 D, and 14.46% of the eyes ended up with a high surge of astigmatism averaging 2.7 ± 1.6 D, Figure 9. Differences among the three categories were significant, with *p* < 0.05.

### 3.3. AI Prediction of Ortho-K Lens Decentration

**Figure 10:**
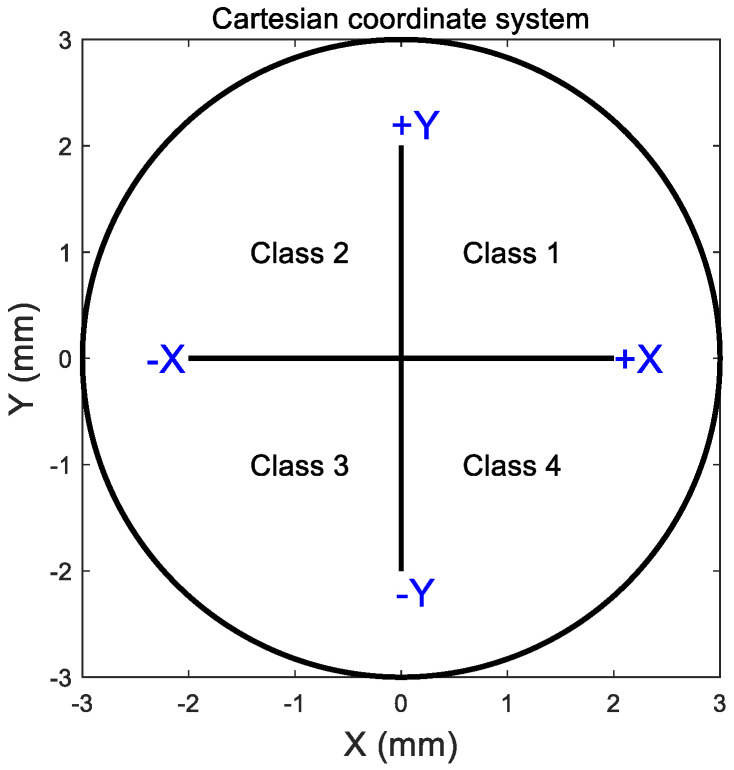
Cartesian coordinate system describing decentration towards the four quarters used in the AI neural network classification.

**Figure 11:**
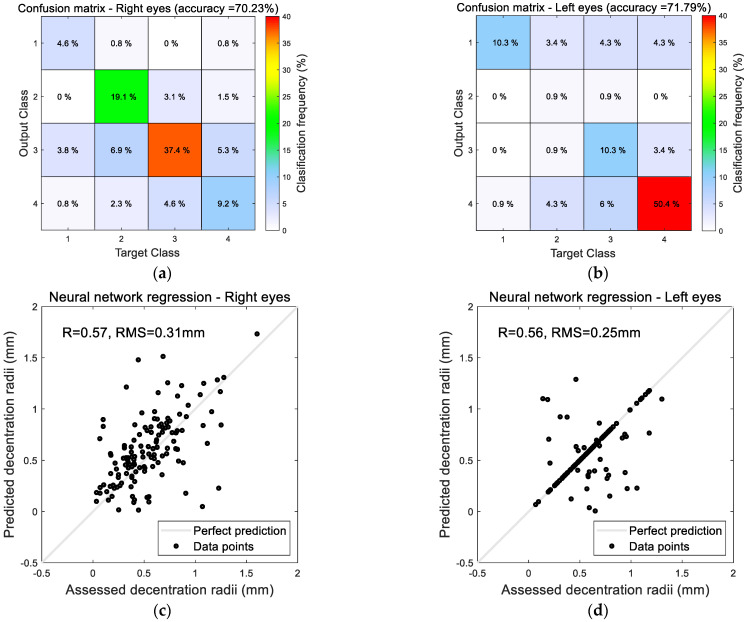
AI-based decentration prediction results towards the four Cartesian quarters as in (**a**,**b**) by radii, predicted as in (**c**,**d**) for right and left eyes, respectively.

In terms of predicting ortho-k lens decentration using the neural network machine learning AI method, classifying right eye decentration in the correct Cartesian quarter was true in 70% of the cases and 72% of left eyes. Using the Cartesian coordinate system as a classifier to point the direction of decentration towards the four quarters, Figure 10, 37.4% of decentrations were predicted in quarter 3 in right eyes (temporal inferior), and 50.4% of decentrations were predicted in quarter 4 in left eyes (temporal inferior). Radii of decentration measured from the centre of the cornea were predicted with RMS of 0.31 mm in right eyes with R = 0.57 to the assessed decentration radii and RMS of 0.25 mm for left eyes with R = 0.56 to the assessed decentration radii, Figure 11.

## 4. Discussion

Decentration in ortho-k contact lens fitting is multifactorial, and some of the causes differ from those found in conventional lenses where the posterior surface base curve has a clear relationship to the corneal shape. In ortho-k contact lens design, the base curve not only takes corneal shape into consideration but also the amount of refractive error to be corrected, whereas the alignment curve is only associated to the corneal shape [45,46]. Thus, two different corneas may have similar peripheral corneal shapes, but the base curve of the ortho-k lens may differ due to the target power requirements. In terms of corneal shape, it has been found that moderate-to-high astigmatism (more than 1.0 D) can cause the decentration of rotationally symmetrical ortho-k lenses [47], whereas spherical corneas usually do not [48]. Additionally, ortho-k wearing patterns are unique in that lenses are typically worn overnight so that acceptable vision is attained lens-free the next day. When conventional RGP lenses are worn, fit assessment (with white light and fluorescein) occurs in the same environment as when the lens is worn during the day. Therefore, eye care practitioners (ECP) can assess the effect of blinking, tear film, and lens movements and reasonably predict the lens’s behaviour during wear. In contrast, with ortho-k lenses, the fit assessment takes place with the patient upright with the eyes open whilst the lens is worn, while the patient is supine with eyes closed during sleep. Unlike conventional lenses, the position of ortho-k lenses that are worn overnight can be influenced by the pressure of the closed eyelid [45] and the patient’s sleeping position, and it has been shown that predicting TZ position on topography maps from open-eye lens assessment is difficult [49]. An added complication is that the pupil position is not always centrally placed compared to the cornea [50,51] and can vary significantly between individuals. It is, therefore, important to understand the effects of decentration and develop methods to predict it to improve fitting outcomes.

Decentration of ortho-k contact lenses leads to a misalignment of the TZ, the central area of the cornea flattened by the base curve, relative to the visual axis. Preferably, for myopia management, the TZ should be centred on the pupil to provide optimal vision correction, whereas, for myopia control, some decentration may be desirable to influence axial growth outcomes. In the current study, more than 50% of the eyes investigated demonstrated significant decentration of the TZ, which can lead to visual disturbances where patients may experience glare, halos, double vision, or ghosting, especially in low-light conditions with larger pupil sizes. 

Zernike polynomial analysis indicates that the low-order aberration terms “piston”, “vertical tilt”, “horizontal tilt”, “vertical astigmatism”, and high-order aberration terms “vertical coma”, “primary spherical aberration”, “vertical secondary astigmatism”, and “vertical quatrefoil” changed significantly as a result of ortho-k wear. Logically, one would expect significant changes only in “piston” and “defocus” terms if lenses were entirely centred, so the other induced HOAs are likely to be a direct effect of decentration.

This study showed that around 60% of the eyes which underwent ortho-k treatment demonstrated a significant increase in astigmatism due to decentration of more than 0.5 mm. Around 14.5% of the eyes recorded increased astigmatism of at least 2 D and up to 12 D in some cases. This indicates that using a rotationally symmetric design in ortho-k lenses may not be ideal in over 50% of cases, and either adding stability features or using toric back ortho-k lens designs may offer an appropriate solution to controlling decentration. 

Regarding the direction of decentration, the current study agrees with the literature [19,20] that most rotationally symmetrical lenses decentre in a temporal inferior direction. Although the average decentration radii were only around 0.65 mm, the range of decentration extended up to 2 mm, with 61% of lenses decentred more than 0.5 mm.

AI can potentially revolutionise ortho-k by improving predictions of lens decentration [52]. Using AI, machine-learning-based algorithms can analyse corneal topography data to detect patterns that lead to decentration, which can then be used to develop methodologies for predicting TZD from corneal shape and target power requirements.

Artificial neural networks are AI machine learning practices that can be used to classify groups and perform regression tasks. In classification, they assign input data to predefined categories, such as the four cardinal quadrants. In regression tasks, they describe the relationship between input and target variables [53], such as the magnitudes of lens decentrations. Neural networks can learn complex patterns and nonlinear relationships within data while optimising the fitting by minimising the fit error during training [54,55]. They consist of interconnected sets of nodes organised in layers: an input layer, a group of hidden layers, and an output layer [37].

One limitation of the current study is that it did not examine the factors that may have caused the decentration, including whether the final fit of the lens was optimal. As discussed in the introduction, many factors can contribute to decentration in ortho-k contact lens wear, and a future study could attempt to obtain tighter control over the fitting outcomes to investigate the effects of decentration more clearly. Neural networks can enhance ortho-k contact lens fitting by improving the prediction of lens decentration, allowing much greater control over the fitting outcomes, whether better centration for myopia management or targeted decentration for specific myopia control effects. An interesting finding was that lens decentration was better AI-predicted for left eyes than right eyes, Figure 11. As ortho-k lenses are worn overnight, when the patient sleeps, such fellow eye differences may connect to the laterality of the sleeping position. It has been shown that there is a link between laxity of the upper lid and the side on which individuals sleep [56], potentially caused by mechanical interaction between the lid and pillow. A study comparing lateral sleep position to IOP showed that subjects were 1.6 times more likely to sleep on the right side, which correlated with a lower IOP in the left eye [57]. As the current study was retrospective, it was not possible to know the preferred sleeping position of the participants. Still, this data will be considered in future work, and both IOP and CCT could be additional inputs to AI-based prediction models. The current study’s findings support the research direction of analysing right and left eyes separately [27,31,51,58,59] so that differences in their characteristics and responses to myopia management or control treatments can be detected.

## 5. Conclusions

Ortho-k lens decentration is common when fitting rotationally symmetrical designs, resulting in HOAs and astigmatism being induced with the magnitude related to the amount of decentration. AI-based algorithms can effectively predict decentration, potentially allowing for much better control over ortho-k fitting and, thus, preferred clinical outcomes.

## Figures and Tables

**Table 1:** The participants selected for the study based on time wearing Ortho-K lenses.

Time of Wear (Days)	Number of Eyes	Age in Years (m ± std)	IOP (mmHg)	CCT (µm)	Pre-Simulated Keratometry (Sim-K) (D)		Post-Simulated Keratometry (Sim-K) (D)		Eye	
					Flat (m ± std)	Steep (m ± std)	Flat (m ± std)	Steep (m ± std)	Right	Left
10 to 100	249	14.10 ± 4.0	15 ± 3	554 ± 33	42.62 ± 1.31	43.96 ± 1.34	41.07 ± 1.33	42.71 ± 1.49	132	117

**Table 2:** Zernike term coefficients pre- and post-ortho-k wear and the statistical evaluation of their change, where (*) indicates a significant change.

Aberrations	$Z_{n}^{m}$	Zernike Term	Mean Pre	STD Pre	Mean Post	STD Post	*p* Pre vs. Post
Low order aberrations	$Z_{0}^{0}$	Piston	−0.9119	0.0742	−0.8770	0.0621	<0.0001 *
	$Z_{1}^{-1}$	Vertical tilt	−0.0001	0.0150	+0.0187	0.0393	<0.0001 *
	$Z_{1}^{1}$	Horizontal tilt	+0.0041	0.0148	−0.0023	0.0203	0.00066 *
	$Z_{2}^{-2}$	Oblique astigmatism	+0.0005	0.0091	+0.0025	0.0131	0.08541
	$Z_{2}^{0}$	Defocus	−0.9384	0.0782	−0.9186	0.0678	0.00979
	$Z_{2}^{2}$	Vertical astigmatism	+0.0362	0.0185	+0.0514	0.0265	<0.0001 *
High order aberrations	$Z_{3}^{-3}$	Vertical trefoil	−0.0039	0.0049	−0.0053	0.0099	0.08019
	$Z_{3}^{-1}$	Vertical coma	−0.0008	0.0081	+0.0074	0.0166	<0.0001 *
	$Z_{3}^{1}$	Horizontal coma	+0.0030	0.0104	+0.0009	0.0070	0.02539
	$Z_{3}^{3}$	Oblique trefoil	+0.0013	0.0046	+0.0018	0.0071	0.40385
	$Z_{4}^{-4}$	Oblique quatrefoil	+0.0007	0.0030	+0.0003	0.0043	0.34585
	$Z_{4}^{-2}$	Oblique secondary astigmatism	−0.0001	0.0032	+0.0013	0.0057	0.00508
	$Z_{4}^{0}$	Primary spherical aberration	−0.0265	0.0058	−0.0415	0.0102	<0.0001 *
	$Z_{4}^{2}$	Vertical secondary astigmatism	+0.0065	0.0067	+0.0161	0.0115	<0.0001 *
	$Z_{4}^{4}$	Vertical quatrefoil	+0.0003	0.0039	−0.0050	0.0054	<0.0001 *

## Data Availability

The data presented in this study are available on request from the corresponding author. The data are not publicly available due to considerations regarding possible future commercialisation.
